# Tolerable glycometabolic stress boosts cancer cell resilience through altered *N*-glycosylation and Notch signaling activation

**DOI:** 10.1038/s41419-024-06432-z

**Published:** 2024-01-15

**Authors:** Shungo Iwamoto, Takashi Kobayashi, Hisatoshi Hanamatsu, Ikuko Yokota, Yukiko Teranishi, Akiho Iwamoto, Miyu Kitagawa, Sawako Ashida, Ayane Sakurai, Suguru Matsuo, Yuma Myokan, Aiyu Sugimoto, Ryo Ushioda, Kazuhiro Nagata, Noriko Gotoh, Kazuki Nakajima, Takashi Nishikaze, Jun-ichi Furukawa, Naoki Itano

**Affiliations:** 1 Graduate School of Life Sciences, Kyoto Sangyo University, Kyoto, Japan; 2 Faculty of Life Sciences, Kyoto Sangyo University, Kyoto, Japan; 3 Department of Orthopaedic Surgery, Faculty of Medicine and Graduate School of Medicine, Hokkaido University, Sapporo, Hokkaido Japan; 4 Institute for Glyco-core Research (iGCORE), Nagoya University, Nagoya, Aichi Japan; 5 JT Biohistory Research Hall, Takatsuki, Osaka, Japan; 6 Division of Cancer Cell Biology, Cancer Research Institute, Institute for Frontier Science Initiative, Kanazawa University, Kanazawa, Ishikawa Japan; 7 Institute for Glyco-core Research (iGCORE), Gifu University, Gifu, Japan; 8 Solutions COE, Analytical & Measuring Instruments Division, Shimadzu Corporation, Kyoto, Japan

**Keywords:** Cancer metabolism, Cancer stem cells, Glycobiology, Stress signalling

## Abstract

Chronic metabolic stress paradoxically elicits pro-tumorigenic signals that facilitate cancer stem cell (CSC) development. Therefore, elucidating the metabolic sensing and signaling mechanisms governing cancer cell stemness can provide insights into ameliorating cancer relapse and therapeutic resistance. Here, we provide convincing evidence that chronic metabolic stress triggered by hyaluronan production augments CSC-like traits and chemoresistance by partially impairing nucleotide sugar metabolism, dolichol lipid-linked oligosaccharide (LLO) biosynthesis and *N*-glycan assembly. Notably, preconditioning with either low-dose tunicamycin or 2-deoxy-D-glucose, which partially interferes with LLO biosynthesis, reproduced the promoting effects of hyaluronan production on CSCs. Multi-omics revealed characteristic changes in *N*-glycan profiles and Notch signaling activation in cancer cells exposed to mild glycometabolic stress. Restoration of *N*-glycan assembly with glucosamine and mannose supplementation and Notch signaling blockade attenuated CSC-like properties and further enhanced the therapeutic efficacy of cisplatin. Therefore, our findings uncover a novel mechanism by which tolerable glycometabolic stress boosts cancer cell resilience through altered *N*-glycosylation and Notch signaling activation.

## Introduction

Metabolic stresses occur in various cancer types owing to the limited availability of nutrients and high metabolic demands. Cancer cells develop multiple adaptive mechanisms to limit these metabolic stresses, increase their metabolic flexibility and resilience, and gain survival and stress resistance by rewiring intracellular metabolic and signaling cascades [1].

Cancer stem cells (CSCs) represent a small subpopulation of self-renewing oncogenic cells that are suggested to be responsible for cancer recurrence and therapeutic resistance. Therefore, they have been considered promising targets for curative therapies in cancer. The importance of metabolic stress in controlling the dynamic interconversion between non-stem cancer cells and CSCs has been highlighted in studies of many solid tumors [2–4]. To date, however, it is not fully understood how metabolic stress responses contribute to the emergence, maintenance, and therapeutic resistance of CSCs.

Aberrant glycosylation, a hallmark of cancer, plays a key role in modulating cancer cell proliferation, metastasis, immune evasion, and multidrug resistance [5–7]. Cancer cells dynamically alter their glycan composition and structure by sensing fluctuations in a wide spectrum of glucose metabolites [8–10]. Emerging evidence has shown that CSCs have different glycosylation profiles than non-stem cancer cells [11–14], suggesting the relevance of glycosylation in the regulation of CSC characteristics.

Hyaluronan (HA) is a linear polysaccharide in the extracellular matrix whose biosynthesis is regulated by three HA synthases (HAS1–3) that link *N*-acetyl-D-glucosamine (GlcNAc) and D-glucuronic acid (GlcUA) [15, 16]. A growing body of evidence has shown that HA accumulation is correlated with poor prognosis in patients with advanced cancers [17–20]. In addition to the importance of HA accumulation in cancer progression, recent metabolomic approaches have revealed that HA also regulates CSC-like properties by reprogramming cellular metabolism coupled with its biosynthesis [21].

In this study, we investigated the possible molecular mechanisms underlying the metabolic regulation of CSCs governed by HA biosynthesis. Total cellular glycomics revealed characteristic changes in *N*-glycan profiles and significantly reduced levels of dolichol lipid-linked oligosaccharides (LLOs) in HA-overproducing breast cancer cells. Owing to the extremely large size of HA, HA biosynthesis consumes large quantities of nucleotide sugar donors, uridine diphosphate (UDP)-GlcNAc and UDP-GlcUA. The current results imply that the alterations in *N*-glycan profiles found in HA-overproducing cancer cells were an adaptation to the limited availability of glycosylation precursors. Intriguingly, preconditioning with either low-dose tunicamycin (TM) or 2-deoxy-D-glucose (2-DG), both of which partially interfere with LLO biosynthesis and *N*-glycan assembly, promoted CSC-like traits and chemoresistance, reasonably reproducing the effects of HA overproduction on CSC regulation. RNA-seq transcriptional analysis revealed activation of the Notch signaling pathway in cancer cells exposed to mild glycometabolic stress, and Notch signaling blockade attenuated mammosphere formation and cisplatin resistance in CSCs. Furthermore, the restoration of *N*-glycan assembly with glucosamine (GlcN) and mannose (Man) supplementation suppressed CSC self-renewal and enhanced the therapeutic efficacy of cisplatin. Altogether, our study provides novel evidence that tolerable glycometabolic stress promotes CSC-like properties and chemoresistance through altered *N*-glycosylation and Notch signaling activation.

## Results

### Total cellular glycomics revealed characteristic changes in *N*-linked glycoforms associated with HA overproduction

**Fig. 1:**
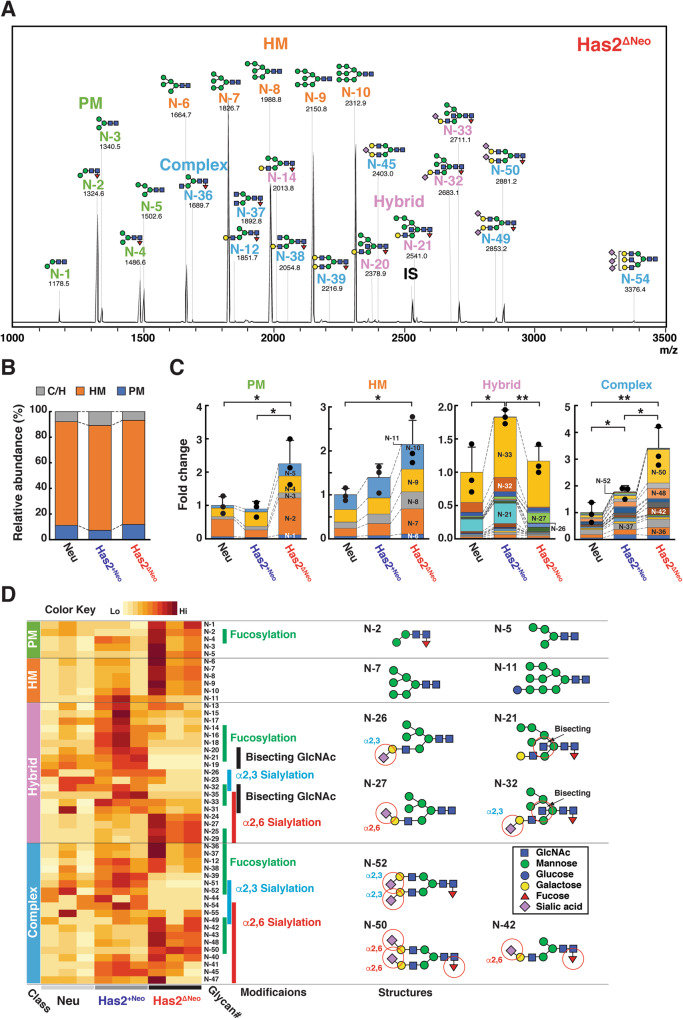
*N*-glycan profiles in mammary carcinoma cell lines with different HA-producing abilities. **A** Typical MALDI-TOF MS spectra of *N*-glycans in HA-high Has2^ΔNeo^ cells. The estimated *N*-glycan structures are shown. IS, internal standard. **B** *N*-glycan composition of parental Neu, HA-low Has2^+Neo^, and HA-high Has2^ΔNeo^ cells. The *N*-glycan composition was expressed as the ratio of each glycan type to the total *N*-glycans. **C** Relative amounts of PM, HM, and C/H-type *N*-glycans. The colors representing each glycoform are listed in Supplementary Table S1. Data are the mean ± SD from *n* = 3. Two-tailed Student’s *t*-test. *p < 0.05, **p < 0.01. **D** Heatmap analysis based on the quantitative glycomic profiles of *N*-glycans. The heatmap represents the normalized abundance of *N*-glycans in Neu, Has2^+Neo^, and Has2^ΔNeo^ cells. The complete structures of the *N*-glycans are shown in Supplementary Table S1. The bars on the right side of the heatmap indicate modifications of the *N*-glycan. Selected glycan structures are shown on the right, with important modifications (red circles).

Integration of omics datasets aids in a comprehensive understanding of the molecular mechanisms underlying CSC regulation. In this study, we initially conducted glycomics to identify the glycoform signature characteristic in HA-overproducing cancer cells. The overall glycoforms were compared among three mammary carcinoma cell lines with different HA-producing abilities: parental Neu cancer cells derived from mammary tumors in mouse mammary tumor virus (MMTV)-Neu transgenic (Tg) mice and two primary breast cancer cell lines, HA-low Has2^+Neo^ and HA-high Has2^ΔNeo^ cells, both of which were derived from Has2 conditional Tg mice carrying the Neu transgene [22]. Total cellular glycomics revealed that the *N*-glycan profile was altered in HA-high Has2^ΔNeo^ cells (Fig. 1A), whereas the profiles were similar in parental Neu and HA-low Has2^+Neo^ cells (Fig. 1B‒D). *N*-glycans are classified into four categories: paucimannose (PM), high-mannose (HM), and complex/hybrid (C/H). The relative amounts of PM-type *N*-glycans, which accounted for approximately 11.1% of the total cellular *N*-glycans in parental Neu cells (Fig. 1B), were significantly increased in HA-high Has2^ΔNeo^ cells (Fig. 1C, D). In parental Neu cells, HM-type glycans, which accounted for approximately 81.0% of total cellular *N*-glycans (Fig. 1B), were predominantly composed of Man_5-9_GlcNAc_2_ glycoforms (Fig. 1C, D). Increased levels of short Man_5-7_GlcNAc_2_ glycoforms were evident in HA-high Has2^ΔNeo^ cells (Fig. 1C, D and Supplementary Table S1), which was indicative of impaired *N*-glycosylation status. The relative amount of hybrid-type *N*-glycan species with bisecting GlcNAc structures decreased in Has2^ΔNeo^ cells (Fig. 1C, D and Supplementary Table S1). The relative amount of complex-type *N*-glycans was significantly elevated in an HA production-dependent manner (Fig. 1C). Of the complex-type *N*-glycans, terminal α2,6-sialylated and core-fucosylated glycan species were enriched in HA-high Has2^ΔNeo^ cells, whereas the relative amount of α2,3-sialylated *N*-glycan species was relatively high in HA-low Has2^+Neo^ cells (Fig. 1C, D and Supplementary Table S1).

### Decreased cellular pools of *N*-glycan precursors in HA-overproducing cancer cells

**Fig. 2:**
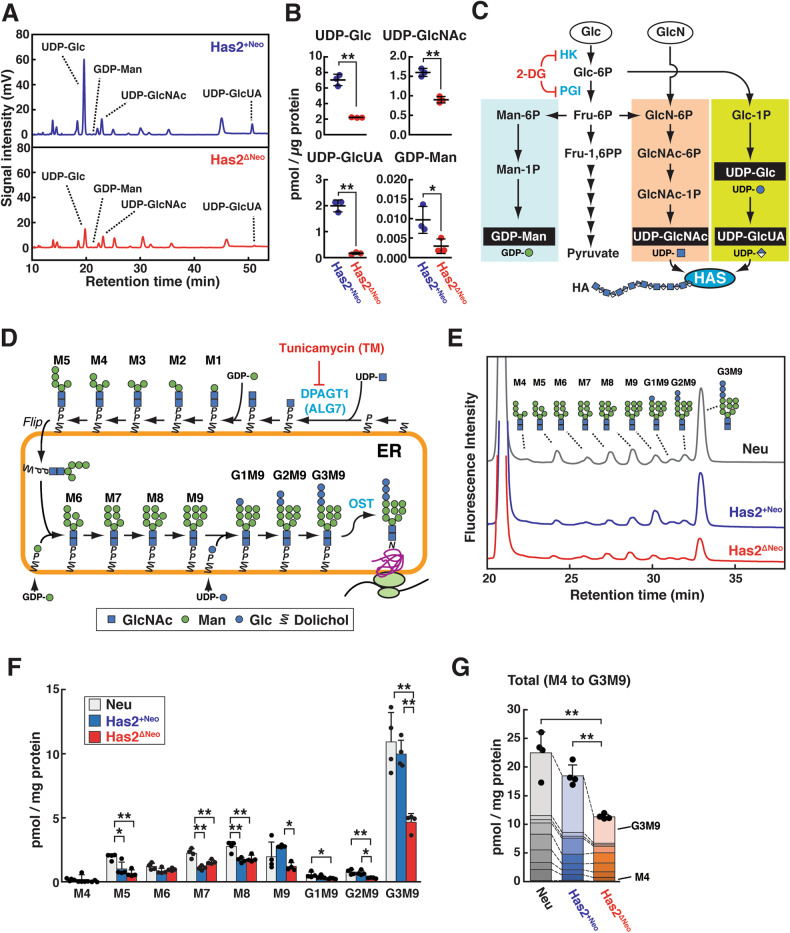
Cellular levels of nucleotide sugars and LLOs in mammary carcinoma cell lines with different HA-producing abilities. **A, B** Ion-pair reversed-phase HPLC profiles (**A**) and cellular levels (**B**) of nucleotide sugars in HA-low Has2^+Neo^ and HA-high Has2^ΔNeo^ cells. Data are the mean ± SD from *n* = 3. Two-tailed Student’s *t*-test. *p < 0.05, **p < 0.01. **C** Schematic diagram of nucleotide sugar biosynthesis. UDP-GlcNAc and GDP-Man are synthesized de novo from a common glycolytic intermediate, fructose-6-phosphate (Fru-6P), in the hexosamine biosynthetic pathway (orange) and GDP-Man biosynthetic pathway (blue), respectively. UDP-GlcUA is produced by a three-step reaction (yellow‒green): conversion of glucose-6-phosphate (Glc-6P) into glucose-1-phosphate (Glc-1P), conversion of Glc-1P into UDP-Glc, and oxidation of UDP-Glc. UDP-GlcNAc and UDP-GlcUA are utilized for HA biosynthesis. **D** Schematic diagram of LLO biosynthesis. The assembly of LLO precursors is initiated by the addition of GlcNAc-phosphate to dolichol-phosphate (Dol-P) from UDP-GlcNAc on the cytosolic face of the ER membrane. After translocation into the ER lumen, the mature form of LLOs, Glc_3_Man_9_GlcNAc_2_-PP-Dol, is synthesized and transferred onto asparagine residues within nascent polypeptide acceptors by the oligosaccharyltransferase (OST) complex. **E‒G** HPLC profiles (**E**) and quantification (**F, G**) of LLO glycans prepared from Neu, Has2^+Neo^, and Has2^ΔNeo^ cells. Data are the mean ± SD from *n* = 4. Tukey’s test. *p < 0.05, **p < 0.01.

HA overproduction may affect protein *N*-glycosylation through the consumption of nucleotide sugars because the biosynthetic pathways of HA and *N*-glycans share the common donor substrate UDP-GlcNAc. Therefore, we measured the cellular levels of nucleotide sugars in HA-low Has2^+Neo^ and HA-high Has2^ΔNeo^ cells using ion-pair reversed-phase HPLC (Fig. 2A). Among the measured nucleotide sugars, the cellular levels of UDP-GlcNAc and UDP-GlcUA were significantly lower in Has2^ΔNeo^ cells than in Has2^+Neo^ cells (Fig. 2B). The decreased intracellular concentrations of UDP-GlcNAc and UDP-GlcUA in HA-overproducing cancer cells are considered to be a consequence of their excessive consumption by HA biosynthesis. Interestingly, intracellular levels of UDP-glucose (UDP-Glc) and guanosine diphosphate (GDP)-Man were approximately 3-fold lower in Has2^ΔNeo^ cells than in Has2^+Neo^ cells (Fig. 2B). As illustrated in Fig. 2C, intracellular pools of nucleotide sugars were comprehensively regulated by glucose metabolism. Therefore, the cellular profiles of major nucleotide sugars suggest a countervailing mechanism by which cells can balance the supply of nucleotide sugars in the event of an imbalance.

Reduced levels of UDP-Glc, UDP-GlcNAc, and GDP-Man, all of which are essential substrates for *N*-glycan precursor biosynthesis (Fig. 2D), may perturb the full assembly of mature LLOs and thereby alter *N*-glycan biosynthesis. Therefore, we investigated whether HA production affects LLO biosynthesis. As expected, HA-high Has2^ΔNeo^ cells had significantly lower amounts of mature Glc_3_Man_9_GlcNAc_2_-PP-Dol than the parental Neu and HA-low Has2^+Neo^ cells (Fig. 2E‒G). Neu and Has2^+Neo^ cells displayed different profiles of immature LLOs without a significant reduction in the levels of mature LLO (Fig. 2E‒G). Collectively, these findings suggest that the reduced level of mature LLO is responsible for the alterations of *N*-glycosylation in HA-high Has2^ΔNeo^ cells.

### Long-term preconditioning with low-dose TM enhances CSC-like traits and chemoresistance

**Fig. 3:**
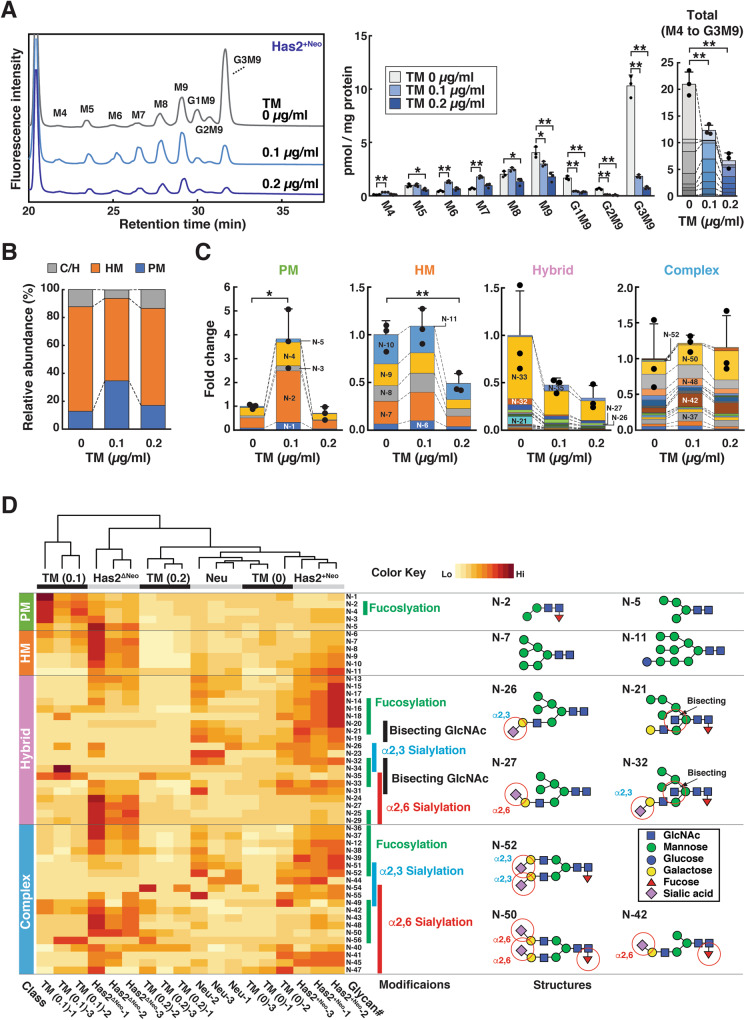
Low-dose TM preconditioning partially interferes with LLO biosynthesis and *N*-glycan assembly. **A** HPLC profiles and cellular levels of LLO glycans. Low-HA Has2^+Neo^ cells were treated with 0.1 or 0.2 μg/ml TM for 8 days and analyzed for cellular LLO levels. Data are the mean ± SD from *n* = 3. Tukey’s test. *p < 0.05, **p < 0.01. **B** *N*-glycan composition of TM-treated and untreated cells. Low-HA Has2^+Neo^ cells were treated with 0.1 or 0.2 μg/ml TM for 8 days and analyzed for *N*-glycan compositions. *N*-glycan composition is expressed as the ratio of each glycan type to the total *N*-glycans. **C** Relative amounts of PM, HM, and C/H-type *N*-glycans. The colors representing each glycoform are listed in Supplementary Table S2. Data are the mean ± SD from *n* = 3. Two-tailed Student’s *t*-test. *p < 0.05, **p < 0.01. **D** Hierarchical clustering analysis based on the quantitative glycomic profiles of *N*-glycans. The hierarchical clustering heatmap represents the normalized abundance of *N*-glycans in the TM-treated and untreated cells. The Neu, Has2^+Neo^, and Has2^ΔNeo^ cell datasets were reused from Fig. 1D. The complete structures of the *N*-glycans are shown in Supplementary Tables S1 and S2. The bars on the right side of the heatmap indicate *N*-glycan modifications. Selected glycan structures are shown on the right, with important modifications (red circles).

Given that HA overproduction has been demonstrated to increase the number of CSC-like cells [21], the above findings led us to hypothesize that excess HA production promotes the transition from non-CSCs to CSCs through a reduction in LLO assembly. To determine whether partial inhibition of LLO assembly by long-term preconditioning with low-dose TM affects CSC conversion, we pretreated HA-low Has2^+Neo^ cancer cells for 8 days with TM at varying concentrations and evaluated their effects on CSC-like properties. Growth inhibitory effects were apparent at higher doses of TM (0.2–1.0 μg/ml) and more than 80% of the Has2^+Neo^ cells underwent apoptosis at 1.0 μg/ml. Therefore, HA-low Has2^+Neo^ cells were treated with 0.2 µg/ml TM or less for up to 8 days. Long-term preconditioning with a low dose of TM (0.1 µg/ml) partially interfered with the assembly of mature LLO in Has2^+Neo^ cells, causing it to reach levels comparable to those of HA-high Has2^ΔNeo^ cells (Fig. 3A). Glycomics and hierarchical clustering analysis also demonstrated that preconditioning with low-dose TM (0.1 µg/ml) shifted the *N*-glycan composition closer to that of Has2^ΔNeo^ cells (Fig. 3B‒D and Supplementary Table S2). In contrast, long-term preconditioning with a relatively high dose of TM (0.2 µg/ml) markedly interfered with the assembly of mature LLO (Fig. 3A) and reduced overall *N*-glycan production (Fig. 3B‒D and Supplementary Table S2).

**Fig. 4:**
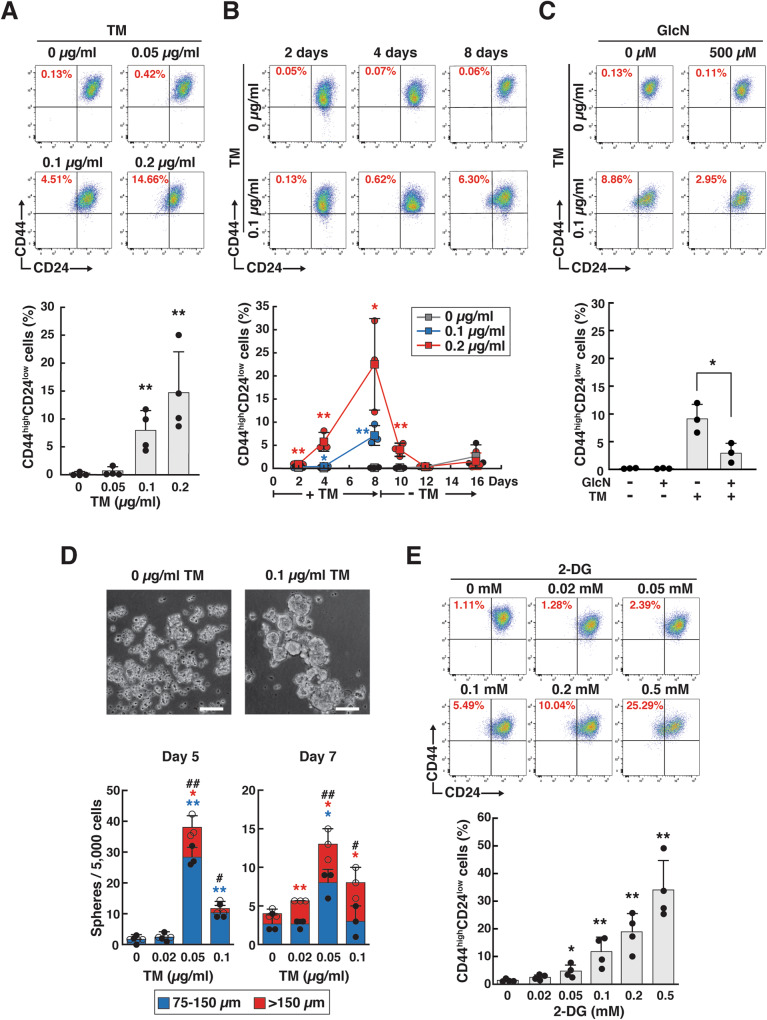
Low-dose TM and 2-DG preconditioning increases the number of CD44^high^/CD24^low^ CSC-like cells. **A, B** FACS analysis of CD44^high^/CD24^low^ CSC-like cells in TM-preconditioned cancer cells. (**A**) Low-HA Has2^+Neo^ cells were treated with different doses of TM for 8 days and analyzed for CD24 and CD44 expression by flow cytometry. Data are the mean ± SD from *n* = 4. Two-tailed Student’s *t*-test. **p < 0.01. (**B**) Low-HA Has2^+Neo^ cells were treated with 0.1 or 0.2 μg/ml TM for 8 days and further cultured in the absence of TM for an additional 8 days. The numbers of CD44^high^/CD24^low^ CSC-like cells were counted at different time points. Data are the mean ± SD from *n* = 3. Two-tailed Student’s *t*-test. *p < 0.05, **p < 0.01. **C** Effects of GlcN supplementation on the number of CD44^high^/CD24^low^ cells. Low-HA Has2^+Neo^ cells were treated with 0.1 µg/ml TM and/or 500 µM GlcN for 8 days and then analyzed for CD24 and CD44 expression by flow cytometry. Data are the mean ± SD from *n* = 3. Two-tailed Student’s *t*-test. *p < 0.05. **D** Mammosphere formation. Has2^+Neo^ cells were treated with low-dose TM for 8 days and cultured for an additional 7 days in ultralow attachment surface 24-well plates with spheroid-forming medium containing the same concentrations of TM. Representative images of mammospheres were taken, and the mammosphere number was counted under a phase-contrast microscope. Scale bar: 100 µm. Data are the mean ± SD from *n* = 3. Two-tailed Student’s *t*-test. *^, #^*p* < 0.05, **^, ##^*p* < 0.01. **E** The effects of 2-DG preconditioning on the number of CD44^high^/CD24^low^ CSC-like cells. Low-HA Has2^+Neo^ cells were treated with different doses of 2-DG for 8 days and analyzed for CD24 and CD44 expression by flow cytometry. Data are the mean ± SD from *n* = 4. Two-tailed Student’s *t*-test. *p < 0.05, **p < 0.01.

Interestingly, low-dose TM preconditioning increased the number of CD44^high^/CD24^low^ CSC-like cells in a dose- and time-dependent manner (Fig. 4A, B). Preconditioning dose-dependently shifted the whole TM-treated cell population toward CD24-low expression (Fig. 4A). Removal of TM from the medium reverted the number of CD44^high^/CD24^low^ CSC-like cells to the levels observed in untreated cells (Fig. 4B), indicating that TM-enhanced CSC-like traits are reversible and not due to clonal selection of TM-resistant cells. GlcN supplementation, which increased the intracellular UDP-GlcNAc pool, partially suppressed the effects of TM preconditioning (Fig. 4C). We evaluated mammosphere formation, reflecting the self-renewal activity, of breast CSCs. Consistent with the increased number of CD44^high^/CD24^low^ CSC-like cells, preconditioning with low-dose TM (0.05 and 0.1 µg/ml) promoted mammosphere formation to a greater extent than no treatment (Fig. 4D).

2-DG suppresses the synthesis of the glycolytic intermediates, Glc-6P and Fru-6P, and interferes with LLO assembly by inhibiting the function of hexokinase (HK) and glucose-6-phosphate isomerase (PGI) (Fig. 2C). Due to its similarity in structure to Man, 2-DG competes with Man metabolism and affects *N*-glycosylation by incorporation into LLOs [23, 24]. 2-DG may also prevent LLO biosynthesis and elongation by inhibiting ATP biosynthesis. To ensure that low-dose 2-DG preconditioning reproduced the promoting effects of HA overproduction on CSC-like traits, we treated Has2^+Neo^ cells for 8 days with various doses of 2-DG and evaluated the number of CD44^high^/CD24^low^ CSC-like cells. Growth inhibitory effects were apparent at 0.2 mM 2-DG and more than 80% of the Has2^+Neo^ cells underwent apoptosis at 2 mM. Therefore, HA-low Has2^+Neo^ cells were treated with a low dose of 2-DG (0.1 mM) for up to 8 days. Consistent with low-dose TM preconditioning, long-term and low-dose 2-DG treatment partially interfered with the assembly of mature LLO in Has2^+Neo^ cells to levels comparable to those observed in HA-high Has2^ΔNeo^ cells (Supplementary Fig. S1A) and shifted the *N*-glycan composition, as in the case of low-dose TM preconditioning (Supplementary Fig. S1B‒D and Supplementary Table S3). Unlike TM preconditioning, 2-DG preconditioning significantly altered the patterns of C/H-type *N*-glycans owing to the de novo production of 2-DG-incorporated glycans (Supplementary Fig. S1C and Supplementary Table S3). As expected, low-dose 2-DG preconditioning increased the number of CD44^high^/CD24^low^ CSC-like cells in a dose-dependent manner (Fig. 4E), thereby supporting the important role of glycometabolic stress in CSC regulation.

**Fig. 5:**
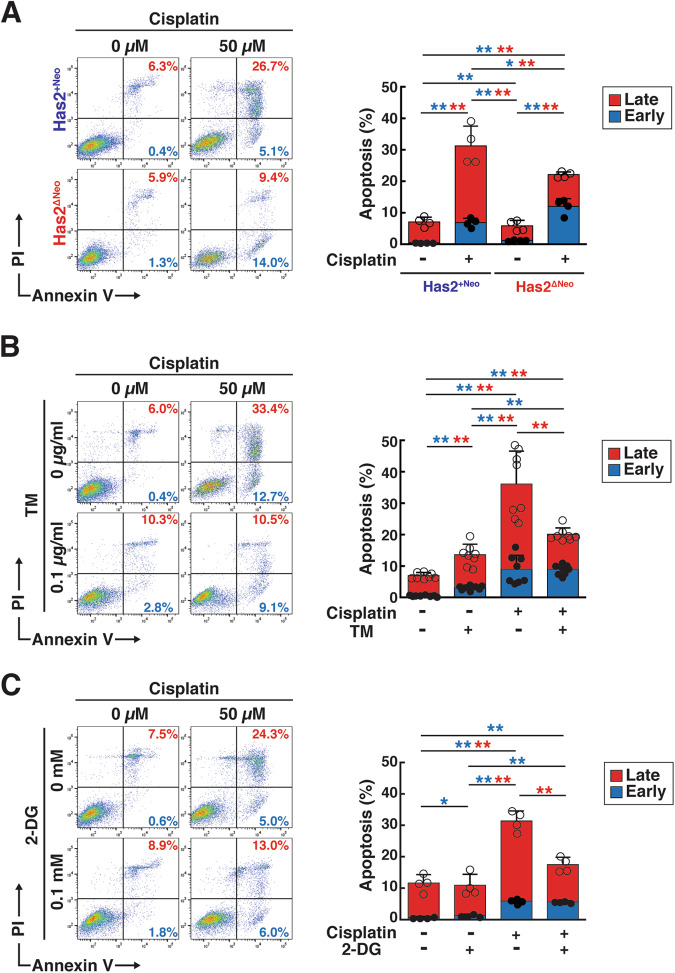
Low-dose TM and 2-DG preconditioning enhances cisplatin resistance. **A** Cisplatin-induced apoptosis in HA-low Has2^+Neo^ and HA-high Has2^ΔNeo^ cells. Has2^ΔNeo^ and Has2^+Neo^ cells were treated with 0-50 µM cisplatin for 16 h. After staining with fluorescent Annexin V and PI, apoptotic cells were analyzed by flow cytometry. Early and late apoptotic cells were represented as Annexin V^+^/PI^-^ and Annexin V^+^/PI^+^ subpopulations, respectively. Data are the mean ± SD from *n* = 4. Two-tailed Student’s *t*-test. *p < 0.05, **p < 0.01. **B, C** Cisplatin-induced apoptosis in Has2^+Neo^ cells pretreated with low-dose TM or 2-DG. HA-low Has2^+Neo^ cells were treated with 0.1 μg/ml TM (**B**) or 0.1 mM 2-DG (**C**) for 8 days. Before staining with fluorescent Annexin V and PI, the cells were treated with 0-50 µM cisplatin for 16 h. Data are mean ± SD from *n* = 8 for (**B**) and *n* = 4 for (**C**). Two-tailed Student’s *t*-test. *p < 0.05, **p < 0.01.

CSCs are highly resistant to conventional chemotherapeutic drugs and are thought to be responsible for tumor recurrence following treatment. HA-high Has2^ΔNeo^ and HA-low Has2^+Neo^ cancer cells were treated with cisplatin, a platinum-based chemotherapeutic drug, and the numbers of early and late apoptotic cells were determined by dual staining with fluorescent Annexin V and propidium iodide (PI). After exposure to 50 μM cisplatin, HA-low Has2^+Neo^ cells displayed marked increases in the number of both early and late apoptotic cells, in contrast to HA-high Has2^ΔNeo^ cells, which were relatively resistant to this dose (Fig. 5A). We examined the effects of preconditioning with TM or 2-DG on chemoresistance. We chose doses of 0.1 μg/ml TM and 0.1 mM 2-DG for combined treatment with cisplatin because these doses only slightly increased the number of apoptotic cells (Fig. 5B, C). Preconditioning with either low-dose TM or 2-DG significantly alleviated cisplatin-induced apoptosis in HA-low Has2^+Neo^ cancer cells (Fig. 5B, C).

### Differential enrichment of gene expression signatures revealed Notch signaling activation under glycometabolic stress

**Fig. 6:**
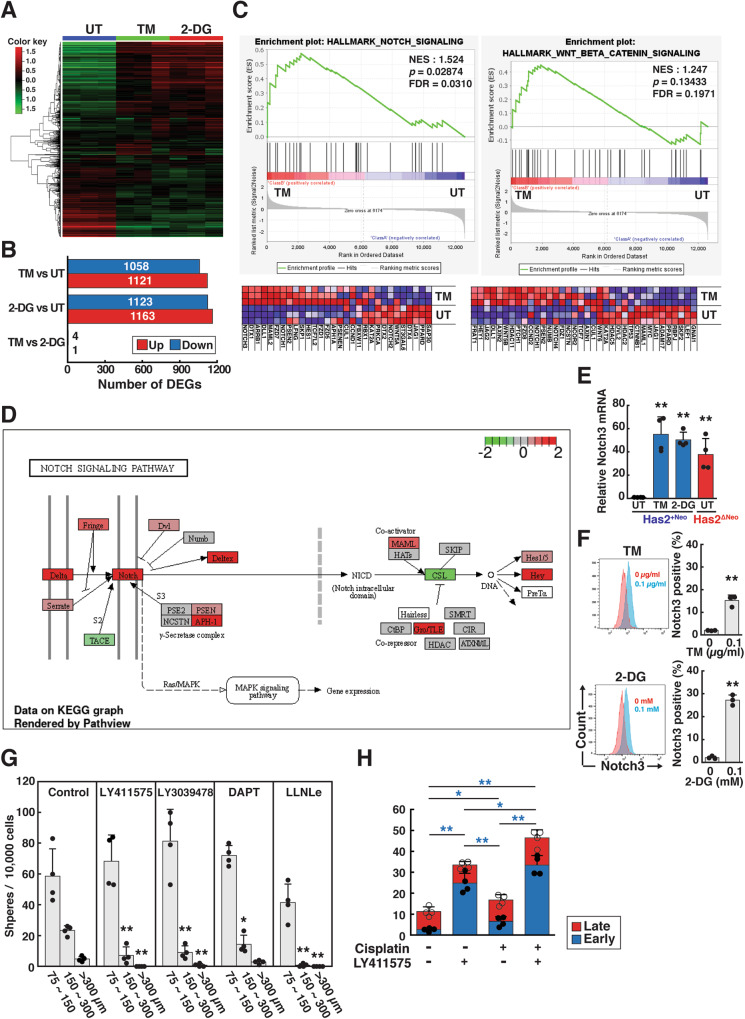
RNA-seq and GSEA of TM- and 2-DG-preconditioned cancer cells. **A** RNA-seq was performed on samples collected after preconditioning HA-low Has2^+Neo^ cells with 0.1 μg/ml TM or 0.1 mM 2-DG. RNA-seq data were compared with those from untreated cells (untreated control, UT). Heatmap of hierarchical clustering indicating DEGs between preconditioned and untreated cells (|log2FC | ≥ 2 and FDR < 0.1). Red and green indicate the upregulated and downregulated genes, respectively. **B** Number of DEGs between samples in each treatment group. The red and blue bars represent the number of upregulated and downregulated genes in each pair, respectively. **C** GSEA hallmark analysis of the pathways significantly upregulated in TM-preconditioned cells versus UT cells. **D** KEGG pathway analysis of RNA-seq data. Red and green indicate the upregulated and downregulated genes, respectively. **E** qRT-PCR analysis of Notch3 expression in Has2^ΔNeo^ and Has2^+Neo^ cells. Has2^+Neo^ cells were treated with 0.1 μg/ml TM or 0.1 mM 2-DG for 8 days and analyzed for Notch 3 expression by qRT‒PCR. The relative expression of Notch 3 mRNA was normalized to that of GAPDH. Data are the mean ± SD from *n* = 4. Two-tailed Student’s *t*-test. **p < 0.01. **F** Induction of cell surface Notch 3 expression by TM- and 2-DG preconditioning. Has2^+Neo^ cells were treated with 0.1 μg/ml TM or 0.1 mM 2-DG for 8 days and analyzed for cell surface Notch 3 expression by flow cytometry. Data are the mean ± SD from *n* = 3. Two-tailed Student’s *t*-test. **p < 0.01. **G** Suppression of mammosphere formation by Notch signaling inhibition. Has2^ΔNeo^ cells were cultured for 7 days in ultra-low attachment surface 24-well plates in spheroid-forming medium containing 50 µM LY411575, 50 µM LY3039478, 20 µM DAPT, or 1 µM LLNLe. The mammospheres were counted under a phase-contrast microscope. Scale bar: 100 µm. Data are the mean ± SD from *n* = 4. Two-tailed Student’s *t*-test. *p < 0.05, **p < 0.01. **H** Enhancement of cisplatin-induced apoptosis by inhibition of Notch signaling. Has2^ΔNeo^ cells were treated with 50 µM LY411575 for 8 days. Before staining with fluorescent Annexin V and PI, the cells were treated with 0-50 µM cisplatin for 16 h. Early and late apoptotic cells were represented as Annexin V^+^/PI^-^ and Annexin V^+^/PI^+^ subpopulations, respectively. Data are the mean ± SD from *n* = 4. Two-tailed Student’s *t*-test. *p < 0.05, **p < 0.01.

We then carried out RNA-seq transcriptional analysis to identify gene expression signatures under glycometabolic stress. Hierarchical clustering of RNA-seq data (16478 genes) was performed for low-dose TM- and 2-DG-preconditioned cells, and the results revealed similar transcriptomic profiles between the two types of preconditioned cells (Fig. 6A). Only five differentially expressed genes (DEGs) were found between TM- and 2-DG-preconditioned cells (Fig. 6B), confirming their similar transcriptomic profiles. Gene set enrichment analysis (GSEA) was conducted to compare TM-preconditioned and untreated cells using a hallmark gene set collection from the Molecular Signatures Database (MSigDB). Strikingly, GSEA functional analysis revealed enrichment patterns of gene signatures related to “Notch signaling” and “WNT/β-catenin signaling” in TM-preconditioned cells (Fig. 6C). Similar trends were observed in the comparison of 2-DG-preconditioned cells with untreated cells (Supplementary Fig. S2). These observations were further highlighted using a hierarchical heatmap (Supplementary Fig. S2). Notch3 was identified as the most upregulated gene in the Notch signaling dataset, and DLL1 and Notch1 were commonly upregulated in both the Notch signaling and WNT/β-catenin signaling datasets. Kyoto Encyclopedia of Genes and Genomes (KEGG) analyses highlighted enhanced Notch signaling in TM-preconditioned cells, with increased expression of Notch receptors, ligands, and target genes such as Hey and Hes (fold-change (log2) cutoff) (Fig. 6D). Largely consistent with the RNA-seq data, qRT‒PCR demonstrated significant upregulation of Notch3 in TM- and 2-DG-preconditioned cells (Fig. 6E). Interestingly, Notch3 was also upregulated in HA-high Has2^ΔNeo^ cells, indicating that these glycometabolic stresses commonly activate the Notch signaling pathway. Consistent with the marked increase in mRNA levels, cell surface Notch3 expression was significantly increased by preconditioning with low-dose TM and 2-DG (Fig. 6F).

Given that activation of the Notch and WNT/β-catenin signaling pathways has been implicated in CSC regulation [25–28], glycometabolic stress is likely to modulate CSC-like properties via these signaling pathways. To investigate the role of Notch signaling in CSC function, we treated Has2^ΔNeo^ cells with Notch signaling inhibitors. Notch inhibitors attenuated mammosphere formation in a dose-dependent manner (Fig. 6G). To address whether Notch signaling also promotes chemoresistance, the synergistic effects of a potent Notch inhibitor (LY411575) and cisplatin on cytotoxicity were evaluated using an apoptosis assay. LY411575 enhanced the cytotoxic effects of cisplatin in Has2^ΔNeo^ cells (Fig. 6H). These findings suggest that glycometabolic stress augments CSC-like traits and chemoresistance, partially through Notch signaling activation.

### GlcN and Man supplementation partially restores *N*-glycan assembly in HA-overproducing cancer cells

**Fig. 7:**
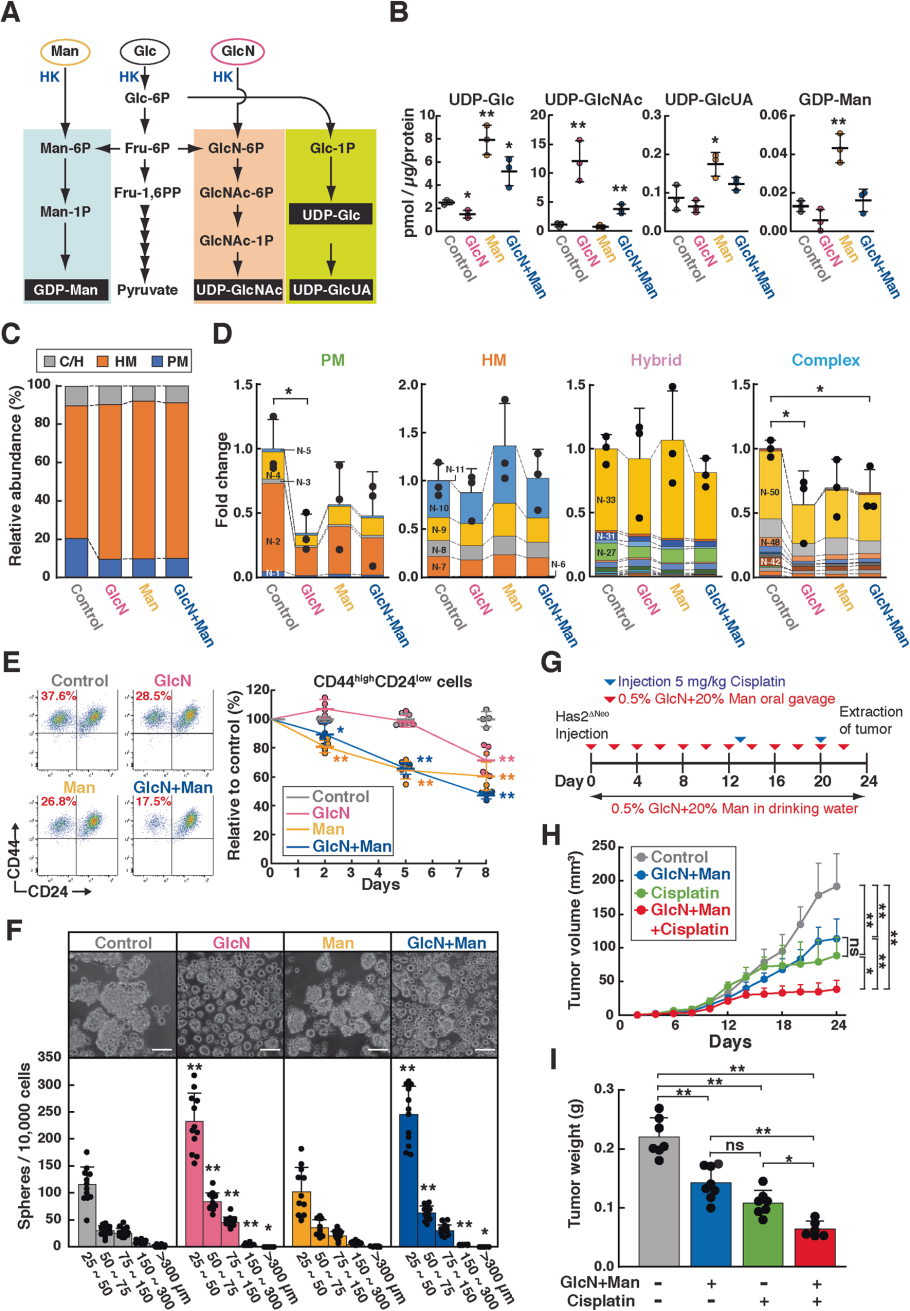
GlcN and Man supplementation suppresses CSC-like properties and augments the therapeutic efficacy of cisplatin. **A** Schematic diagram of nucleotide sugar biosynthesis. **B** Cellular levels of nucleotide sugars. Has2^ΔNeo^ cells were supplemented with 500 μM GlcN and/or 20 mM Man for 24 h and analyzed for cellular levels of nucleotide sugars using ion-pair reversed-phase HPLC. Data are the mean ± SD from *n* = 3. Two-tailed Student’s *t*-test. *p < 0.05, **p < 0.01. **C, D** *N*-glycan composition (**C**) and relative amounts of PM, HM, and C/H-type *N*-glycans (**D**). Has2^ΔNeo^ cells were supplemented with 500 μM GlcN and/or 20 mM Man for 8 days and analyzed for *N*-glycan composition using MALDI-TOF-MS. Colors representing each glycoform are listed in Supplementary Table S4. Data are the mean ± SD from *n* = 3. Two-tailed Student’s *t*-test. *p < 0.05. **E** Effects of GlcN and Man supplementation on the number of CD44^high^/CD24^low^ CSC-like cells. Has2^ΔNeo^ cells were supplemented with 500 μM GlcN and/or 20 mM Man for 8 days and analyzed for CD24 and CD44 expression by flow cytometry. Data are the mean ± SD from *n* = 4. Two-tailed Student’s *t*-test. *p < 0.05, **p < 0.01. **F** Mammosphere formation. Has2^ΔNeo^ cells were supplemented with 500 μM GlcN and/or 20 mM Man for 8 days and cultured for an additional 7 days in ultralow attachment surface 24-well plates with spheroid-forming medium containing the same concentrations of GlcN and/or Man. Representative images of mammospheres were obtained, and the mammosphere number was counted under a phase-contrast microscope. Scale bar: 100 µm. Data are the mean ± SD from *n* = 12. Two-tailed Student’s *t*-test. *p < 0.05, **p < 0.01. **G** Schematic representation of the animal experiment process. **H** Tumor growth of Has2^ΔNeo^ cells in BALB/c nude mice following GlcN and Man supplementation. Tumor sizes were measured at the indicated time points for 24 days, and tumor volumes were calculated as described in the Methods section (*n* = 3-4 mice per group). Tukey’s test. *p < 0.05, **p < 0.01. **I** Tumor weight at the end of the experiments (*n* = 3-4 mice per group). Tukey’s test. *p < 0.05, **p < 0.01.

Reduced nucleotide sugar pools could account for the alterations in *N*-glycan composition in HA-high Has2^ΔNeo^ cells. To address this possibility, cellular pools of nucleotide sugars were analyzed 24 h after the exposure of Has2^ΔNeo^ cells to exogenous GlcN and/or Man (Fig. 7A). GlcN supplementation significantly increased the cellular UDP-GlcNAc pool (Fig. 7B). Moreover, the cellular levels of UDP-Glc, UDP-GlcUA, and GDP-Man were significantly elevated when Has2^ΔNeo^ cells were exposed to high concentrations of exogenous Man (Fig. 7B). The cellular levels of UDP-GlcNAc or GDP-Man were much higher with each treatment alone than with the combined supplementation of GlcN and Man, suggesting that the combined supplementation may resolve the unbalanced accumulation of each nucleotide sugar by properly processing *N*-glycan assembly. *N*-glycan profiles were then analyzed after the exposure of Has2^ΔNeo^ cells to exogenous GlcN and/or Man. The altered *N*-glycan composition in HA-high Has2^ΔNeo^ cells was partially restored upon increases in the levels of cellular UDP-GlcNAc and GDP-Man (Fig. 7C, D). PM-type *N*-glycans, whose levels were elevated in Has2^ΔNeo^ cells, were reduced by GlcN and Man supplementation (Fig. 7C, D). Similarly, GlcN and Man supplementation reduced the proportion of whole complex-type *N*-glycans (Fig. 7D). Notably, these monosaccharides significantly suppressed the synthesis of terminal α2,6-sialylated glycan species (Supplementary Table S4), which was the opposite response to the effects of HA overproduction and low-dose TM preconditioning. Overall, these results suggest that supplementation with GlcN and Man partially restores *N*-glycan assembly in HA-overproducing cancer cells.

### GlcN and Man supplementation suppressed CSC-like properties and augmented the therapeutic efficacy of cisplatin

We then questioned whether restoration of *N*-glycan assembly with GlcN and Man supplementation could suppress CSC-like properties in HA-overproducing cancer cells. To determine if this was the case, we measured the number of CD44^high^/CD24^low^ CSC-like cells after exposure of Has2^ΔNeo^ cells to exogenous GlcN and/or Man. Treatment with GlcN or Man reduced the number of CD44^high^/CD24^low^ CSC-like cells in a time-dependent manner (Fig. 7E). Notably, combined supplementation with GlcN and Man had a greater effect on reducing the number of CSC-like cells. Consistent with the reduced number of CSC-like cells, combined supplementation with GlcN and Man significantly reduced the size of mammospheres compared with that of untreated cells (Fig. 7F). With respect to mammosphere formation, GlcN treatment alone had a strong inhibitory effect, which is in line with a previous study [29]. Since GlcN and Man reversed the CSC-like traits that had been promoted by HA overproduction, we finally investigated whether treatment with these hexoses could also affect tumor growth. Has2^ΔNeo^ cells that had been exposed to GlcN and Man were subcutaneously injected into immunodeficient mice, and the tumor-bearing mice were administered the same hexoses both freely in drinking water and three times a week by oral gavage (Fig. 7G). The combined administration of GlcN and Man significantly inhibited tumor growth and further augmented the therapeutic efficacy of cisplatin (Fig. 7H, I).

## Discussion

This study provides novel mechanistic insights into the molecular basis of metabolic CSC regulation based on the following evidence: (i) chronic metabolic stress triggered by HA production augmented CSC-like traits and chemoresistance by partially impairing nucleotide sugar metabolism, LLO biosynthesis and *N*-glycan assembly; (ii) low-dose and long-term preconditioning with either TM or 2-DG, which partially interfered with LLO biosynthesis and *N*-glycan assembly, enhanced CSC-like traits, and chemoresistance; (iii) low-dose TM and 2-DG preconditioning activated the Notch signaling pathway, and Notch signaling inhibition attenuated mammosphere formation and cisplatin resistance of CSCs; and (iv) restoration of *N*-glycan assembly with GlcN and Man supplementation suppressed CSC-like characteristics and further augmented the therapeutic efficacy of cisplatin. These findings support the idea that tolerable glycometabolic stress boosts cancer cell resilience through altered *N*-glycosylation and Notch signaling activation (Supplementary Fig. S3).

Cancer cells are exposed to persistent metabolic stress because they require a large and continuous nutrient supply to support high metabolic demands. In a cellular environment where the glucose supply is insufficient, cancer cells promote their survival and evasion of apoptosis through the activation of AMPK, phosphorylation of AKT, and production of reactive oxygen species as their adaptive response [30, 31]. The current study further demonstrated that *N*-glycosylation alterations under tolerable glycometabolic stress activated the Notch and WNT/β-catenin signaling pathways, suggesting the particular importance of protein *N*-glycosylation for the regulation of these stemness-related signaling pathways.

*N*-glycosylation is an important post-translational modification for the proper folding, secretion, structural integrity, dynamics, and function of many membrane-bound and secreted proteins. Thus, changes in *N*-glycosylation patterns profoundly influence diverse cellular signals and gene expression involved in cell growth, survival, and stemness. Analysis of the glycomic datasets in this study revealed that HA overproduction and low-dose TM preconditioning commonly altered *N*-glycan profiles, primarily due to elevated levels of unconventional PM- and short HM-type *N*-glycan species, enrichment of terminal α2,6-sialylated and core-fucosylated complex-type *N*-glycan species, and decreased levels of hybrid-type *N*-glycan species with bisecting GlcNAc structure.

PM-type *N*-glycans with a simple structure have traditionally been considered *N*-glycans characteristic of lower organisms. However, recent bioanalytical and immunohistochemical studies have shown that they are a distinct type of *N*-glycan that may play a role in many biological processes in mammals, including those related to immunity, development, and cancer [32–34]. Interestingly, PM-type *N*-glycans are restricted to certain pathophysiological conditions such as cancer and specific stem cell populations [35–37]. A monoclonal Mannitou antibody that is highly specific for the trimannosidic PM structure stains a subpopulation of cytokeratin-positive cancer cells that may represent CSCs.

Increased levels of short Man_5-7_GlcNAc_2_ glycoforms in HM-type *N*-glycans were evident in HA-overproducing and low-dose TM-preconditioned cancer cells. The endoplasmic reticulum (ER) degradation-enhancing α-mannosidase-like (EDEM) family participates in ER-associated protein degradation (ERAD) by trimming the Man_9_GlcNAc_2_ glycoform to Man_5-7_GlcNAc_2_ glycoforms [38]. Moreover, the Man_8_GlcNAc_2_ glycoform is normally translocated to the Golgi apparatus and is progressively trimmed to the Man_5_GlcNAc_2_ glycoform via α-mannosidase activity. Thus, consecutive accumulation of Man_5-7_GlcNAc_2_ glycoforms may be due to the relative inefficiency of *N*-glycosylation processes in both the ER and Golgi apparatus.

Several lines of evidence have demonstrated that α2,6 sialylation is essential for the establishment and maintenance of CSCs [11, 13]. ST6GAL1, which is the predominant sialyltransferase responsible for the α2,6 sialylation of *N*-glycans, plays a crucial role in driving pancreatic CSCs. In this study, we found that both HA overproduction and low-dose TM preconditioning significantly increased terminal α2,6-sialylated and core-fucosylated *N*-glycan species, implying that this modification may be critical for the enhancement of CSC-like traits. The current RNA-seq data indicated no significant changes in the gene expression levels of three α2,3-sialyltransferases, ST3GAL3, ST3GAL4, and ST3GAL6, and α2,6-sialyltransferase, ST6GAL1, all of which are known to be involved in the synthesis of terminal sialylated *N*-glycan species in untreated and TM-preconditioned cancer cells (Supplementary Table S5). Therefore, the gene expression of these sialyltransferases does not appear to be the primary cause of changes in altered sialylation patterns. The presence of bisecting GlcNAc in *N*-glycans interferes with the catalytic activity of sialyltransferases [39] and α2,6 sialylation is less affected by the bisecting GlcNAc structure than α2,3 sialylation [40]. Thus, a moderate reduction in the bisecting GlcNAc structure may preferentially increase the ratio of α2,6-sialylated *N*-glycans in cancer cells exposed to mild glycometabolic stress.

This study highlighted the prominent role of protein *N*-glycosylation in integrating metabolic sensing and CSC signaling pathways. Multi-omics successfully identified a glycoform signature common to cancer cells exposed to mild glycometabolic stress, and elucidated the mechanism by which altered *N*-glycosylation promotes CSC-like properties and chemoresistance through activation of the Notch signaling pathway. The current study further provided evidence that restoration of *N-*glycan assembly through combined supplementation with GlcN and Man significantly reduces CSC-like traits and exacerbates cisplatin-induced tumor suppression. Therefore, this metabolism-based cancer treatment could be a novel strategy for reducing therapy-resistant CSC populations.

## Materials and Methods

### Cell culture

Parental Neu, HA-low Has2^+Neo^, and HA-high Has2^ΔNeo^ primary breast carcinoma cells were established and cultured as previously described [41]. Primary mammary cancer cells were grown in Dulbecco’s modified Eagle’s medium (DMEM; Nacalai Tesque, Kyoto, Japan) containing 10% fetal bovine serum (FBS) under the standard culture conditions of a humidified atmosphere of 95% air and 5% CO_2_ at 37 °C.

### Inhibitor treatment and monosaccharide supplementation

Has2^+Neo^ and Has2^ΔNeo^ cells were seeded at 3.3 × 10^5^ cells/dish into 60 mm cell culture dishes and cultured at 37 °C and 5% CO_2_ for 24 h in DMEM containing 10% FBS. For glycosylation inhibition, Has2^+Neo^ cells were treated with 0-2.0 μg/ml TM (Sigma‒Aldrich, St. Louis, MO) or 0-5 mM 2-DG (Fujifilm Wako Chemicals, Osaka, Japan) for up to 8 days. To inhibit Notch signaling, Has2^ΔNeo^ cells were treated with the Notch signaling inhibitors, 50 µM LY411575 (Selleck Chemicals LLC, Houston, TX), 50 µM LY3039478 (Selleck Chemicals LLC), 20 µM DAPT (Fujifilm Wako Chemicals), and 1 µM LLNLe (Abcam, Cambridge, UK) for 7 days. For monosaccharide supplementation, Has2^ΔNeo^ cells were exposed to 500 μM GlcN and/or 20 mM Man for 8 days.

### Quantitative analysis of *N*-glycans by matrix-assisted laser desorption/ionization-time of flight mass spectrometry (MALDI-TOF MS)

Glycoproteins were extracted as described previously [42]. Briefly, cells were washed 6 times with cold PBS and harvested by scraping in cold PBS. The cell lysate was centrifuged at 500 × g for 5 min at 4 °C. The cell pellet was used for *N*-glycan analysis. One hundred micrograms of cellular protein was subjected to reductive alkylation using Tris (2-carboxyethyl) phosphine (TCEP) and iodoacetamide. After reductive alkylation, a 4-fold volume of ethanol was added to the reaction mixture and incubated at -30 °C for 3 h. The denatured protein was dissolved in ammonium bicarbonate buffer, followed by trypsin digestion and peptide-*N*-glycosidase F digestion, as previously reported [42, 43]. Released *N*-glycan analysis was performed using the glycoblotting method combined with aminolysis-SALSA [44, 45]. The purified glycans were analyzed by MALDI-TOF MS using 2,5-dihydroxybenzoic acid (DHB; Sigma‒Aldrich) as a matrix. The relative amount of each glycan was analyzed using Cluster 3.0 software. A heatmap with hierarchical clustering was acquired using the heatmap function in R, version 4.2.2.

### UDP-sugar quantification by ion-pair reversed-phase HPLC

UDP-sugars were quantified using ion-pair reversed-phase HPLC as previously described [46, 47].

### LLO analysis

LLOs were extracted and analyzed as previously described, with some modifications [48, 49]. Briefly, cells were washed twice with PBS and harvested by scraping in cold PBS. The cell lysate was centrifuged at 500 × g for 5 min at 4 °C. The cell pellet was stored at −80 °C. The frozen cell pellet was resuspended in 1 ml of 100 mM Tris-HCl (pH 7.5) containing 4 mM MgCl_2_. Two milliliters of methanol followed by 3 ml of CHCl_3_ were added to the cell suspension. The mixture was then shaken at room temperature for 60 min. After centrifugation at 2,000 × g for 15 min at 4 °C, three phases (buffer/methanol upper phase, lower CHCl_3_ phase, and interphase) were separated. LLOs were then recovered from both the lower CHCl_3_ phase and the CHCl_3_/methanol/water (10:10:3) extracts of the interphase materials. The LLOs in the organic phase were lyophilized, and the oligosaccharides (LLO glycans) were released by mild acid hydrolysis in 1 ml of HCl containing 50% isopropanol for 30 min at 100 °C. The released LLO glycans were lyophilized and resuspended in 1 ml of water. Water-soluble oligosaccharides were purified using a Spelco ENVI-Carb column (Sigma-Aldrich) [50]. The purified LLO glycans were labeled with 2-aminopyridine (PA, Fujifilm Wako Chemicals) and further purified using a Monospin Amide column (GL Sciences Inc., Tokyo, Japan). PA-labeled glycans were analyzed by size-exclusion HPLC using a Shodex NH2P-50 4E column (4.6 × 250 mm; Resonac Corp. Tokyo, Japan). Elution was performed using two solvent gradients: solvent A (93% acetonitrile and 7% 50 mM ammonium acetate) and solvent B (20% acetonitrile and 80% 50 mM ammonium acetate). The gradient program was as follows: 0-5 min, isocratic 3% solvent B; 5–8 min, 3-33% solvent B; 8-40 min, 33–71% solvent B; 40-60 min, 71-3% solvent B; 60-90 min, isocratic 0% solvent B. The flow rate was 0.8 ml/min, and the column temperature was room temperature. The fluorescence of the labeled glycans was detected at 310 nm excitation and 380 nm emission wavelengths. PA-labeled glycans were quantitated based on the fluorescence intensity of PA-labeled standards, PA-M3B and PA-M5B, purchased from Masuda Chemical Industry Co. Ltd. (Tokushima, Japan).

PA-labeled LLO glycans in each HPLC peak were identified by MALDI-TOF MS using an Autoflex Speed instrument (Bruker Daltonics, Billerica, MA) (Supplementary Fig. S4). Samples were prepared by spotting 1.0 μl of glycan solution onto the target plate (ground steel) together with 1.0 μl of matrix solution (20 mg/ml DHB) and 1.0 μl of 20 mM ammonium sulfate to shift the signal to [M + H]^+^. After drying the sample mixture, the glycans were analyzed in the positive reflection mode. Representative MS spectral data were analyzed and annotated using GlycoWorkbench software (https://code.google.com/archive/p/glycoworkbench/) [51].

### Flow cytometric analysis

Flow cytometric analysis was performed as previously described [47]. To identify CSC-like populations, cells were stained with PE-conjugated anti-CD44 (eBioscience, 12-0441-82, San Diego, CA) and FITC-conjugated anti-CD24 antibodies (eBioscience, 11-0241-82). To detect cell surface Notch3, the cells were stained with a PE-conjugated anti-Notch3 antibody (BioLegend, HMN3-133, San Diego, CA). The distribution of labeled cells was identified by FACS Melody (BD Biosciences, Franklin Lakes, NJ) and analyzed using FlowJo software (BD Biosciences).

### Mammosphere formation assay

The mammosphere formation assay was performed as previously described [52]. For inhibitor treatment, Has2^+Neo^ cells were pretreated with 0.05 or 0.1 µg/ml TM for 8 days. For monosaccharide supplementation, Has2^ΔNeo^ cells were cultured in medium with or without 500 µM GlcN and/or 20 mM Man for 8 days. After treatment with TM or monosaccharides, aliquots of 5,000 Has2^+Neo^ cells or 10,000 Has2^ΔNeo^ cells were seeded into 24-well ultralow attachment plates (Corning, Corning, NY) in serum-free minimal essential medium Ham’s F-12 supplemented with 20 ng/ml bFGF (Fujifilm Wako Chemicals), 20 ng/ml EGF (Miltenyi Biotec, Bergisch Gladbach, Germany), and B27 (Thermo Fisher Scientific, MA) (spheroid-forming medium). The cells were further cultured in medium containing the same concentrations of TM or monosaccharides for 7 days; the medium was replenished with fresh medium every 2-3 days. At the end of culture, the number of spheres with diameters greater than 25 µm was counted under a phase-contrast microscope.

### Apoptosis assay

The apoptosis assay was performed by Annexin V-FITC and PI staining using a MEBCYTO apoptosis kit (MBL Co., Ltd., Nagoya, Japan) according to the manufacturer’s instructions. The distribution of labeled cells was identified using FACS Melody (BD Biosciences) and analyzed using FlowJo software.

### qRT‒PCR

Total RNA was extracted from breast cancer cells using the RNeasy Mini Kit (Qiagen, Germantown, MD), according to the manufacturer’s instructions. Reverse transcription was performed by random priming using the PrimeScript RT Reagent Kit (Takara Bio) according to the manufacturer’s instructions. qRT‒PCR was performed on a QuantStudio3 Flex Real-time PCR system (Thermo Fisher Scientific) with a TaqMan gene expression assay of Notch 3 (Mm00435270_m1, Applied Biosystems, Foster City, CA) and Probe qPCR Mix (Takara Bio). Relative amounts of GAPDH mRNA were measured using TaqMan rodent GAPDH control reagents (Thermo Fisher Scientific). Relative mRNA expression was analyzed using the comparative Ct method and normalized to GAPDH expression.

### RNA‒seq analysis

Total RNA was extracted from Has2^+Neo^ and TM- and 2-DG-preconditioned cells using the RNeasy Mini Kit (Qiagen), according to the manufacturer’s instructions. The purity and concentration of RNA samples were measured using a NanoDrop 2000c instrument (Thermo Fisher Scientific). The RNA integrity number (RIN) was evaluated using an Agilent 2100 Bioanalyzer (Agilent, Santa Clara, CA). Quality-controlled RNA samples with an RIN score of 7 or greater were then subjected to library preparation. cDNA sequencing libraries were prepared using a NEBNext^®^ Poly(A) mRNA Magnetic Isolation Module (for PolyA selection, New England Biolabs, Ipswich, MA) and a NEBNext^®^ UltraTMII Directional RNA Library Prep Kit (for strand-specific libraries, New England Biolabs). RNA sequencing was performed in paired-end 150 bp mode using a NovaSeq 6000 sequencer (Illumina, San Diego, CA) by Rhelixa Co., Ltd. (Tokyo, Japan).

### Analyses of transcriptional data

The reads from RNA-seq data were qualified using FastQC 0.11.7 and filtered using Trimmomatic 0.38 [53, 54]. The read count data were normalized to transcripts per million (TPM), and gene expression was measured using the RaNA-seq cloud platform (https://ranaseq.eu/) [55]. The DEG analysis was conducted with the integrated differential expression and pathway (iDEP) online platform (ver. 0.94) (iDEP Platform http://bioinformatics.sdstate.edu/idep/) [56]. The DESeq2 algorithm was used to filter the DEGs. Genes with |log2FC | ≥ 2 and FDR < 0.1 (false discovery rate (FDR)) were defined as DEGs. Pathway enrichment analyses of DEGs were conducted using the KEGG (https://www.genome.jp/kegg/) metabolic pathway database based on iDEP.

### GSEA

GSEA was performed using a hallmark gene set collection from MSigDB and GSEA software (version: 4.2.3; http://software.broadinstitute.org/gsea/downloads.jsp) [57, 58]. GSEA results were evaluated based on the normalized enrichment score (NES).

### Animal experiment

Has2^ΔNeo^ cells (5.0 ×10^5^ cells) were precultured in DMEM with or without 500 µM GlcN and 20 mM Man for 8 days and injected bilaterally into the fourth mammary fat pads of BALB/c nude mice (three–four animals per group, 8-week-old female, CLEA Japan, Inc., Tokyo, Japan). The mice were given 0.5% (w/v) GlcN and 20% (w/v) Man as drinking water and 100 µL of the same drinking water by oral gavage every other day. Tumor size was measured every 2 days using a digital caliper. The tumor volume was calculated using the following formula: length × width^2^ × 0.5. Cisplatin (5 mg/kg in 200 µL) was injected intraperitoneally at the indicated times. Tumors were excised 24 days after inoculation with cancer cells. Animal care and all experimental procedures were performed in biosafety level 2 animal facilities according to established guidelines approved by the Kyoto Sangyo University Ethics Committee.

### Statistical analysis

Statistical analysis was performed using IBM SPSS Statistics 22 software. All experiments were performed at least three times. The results are reported as the mean ± standard deviation (SD). A significant difference was confirmed when the *P* value < 0.05.

### Supplementary information

Reproducibility Checklist

Supplementary Table S1

Supplementary Table S2

Supplementary Table S3

Supplementary Table S4

Supplementary Table S5

Supplementary Figure legends

Supplementary Figure S1

Supplementary Figure S2

Supplementary Figure S3

Supplementary Figure S4

## Data Availability

All data generated or analyzed during this study are included in this published article and its supplementary information files. The RNA-seq data for this study have been deposited in the DDBJ Sequenced Read Archive under accession number DRA015712.
