# Supplementary material for: Tolerable glycometabolic stress boosts cancer cell resilience through altered N-glycosylation and Notch signaling activation
Source: Cell Death Dis. 2024 Jan 15;15(1):53. doi: 10.1038/s41419-024-06432-z (PMC10789756; doi:10.1038/s41419-024-06432-z)
Supplement: Supplementary file 7 — Supplementary Figure legends [file 41419_2024_6432_MOESM7_ESM.docx]

**Supplementary Figure Legends**

**Supplementary Fig. S1.** **Low-dose 2-DG preconditioning partially interferes with LLO biosynthesis and *N*-glycan assembly.**

**A** HPLC profiles and cellular levels of LLO glycans. Low-HA Has2^+Neo^ cells were treated with 0.1 mM 2-DG for 8 days and analyzed for cellular LLO levels. Data are the mean ± SD from *n* = 3. Two-tailed Student’s *t*-test. ***p* < 0.01. **B** *N*-glycan compositions of 2-DG-treated and untreated cells. Low-HA Has2^+Neo^ cells were treated with 0.1 mM 2-DG for 8 days and analyzed for *N*-glycan compositions. The *N*-glycan composition is expressed as the ratio of each glycan type to the total *N*-glycans. **C** Relative amounts of PM, HM, and C/H type *N*-glycans. The colors representing each glycoform are listed in Supplementary Table S3. Data are the mean ± SD from *n* = 3. Two-tailed Student’s *t*-test. ***p* < 0.01. **D** Hierarchical clustering analysis based on the quantitative glycomic profiles of *N*-glycans. The hierarchical clustering heatmap represents the normalized abundance of *N*-glycans in 2-DG-treated and untreated cells. The Neu, Has2^+Neo^, Has2^ΔNeo,^ and TM-preconditioned cell datasets were reused from Figs. 1D and 3D. The complete structures of the *N*-glycans are shown in Supplementary Tables S1‒S3. The bars on the right side of the heatmap indicate modifications of the *N*-glycan. Selected glycan structures are shown on the right with important modifications (red circle).

**Supplementary Fig. S2. RNA-seq and GSEA of TM- and 2-DG-preconditioned cancer cells.**

**A** GSEA hallmark analysis of the pathways significantly upregulated in 2-DG-preconditioned cells versus UT. RNA-seq was performed on samples collected after preconditioning HA-low Has2^+Neo^ cells with 0.1 mM 2-DG. The RNA-seq data were compared to those from untreated cells (untreated control, UT). **B** Top 20 enriched genes for “Notch signaling” and “WNT/β-catenin signaling” in TM- and 2-DG-preconditioned cells.

**Supplementary Fig. S3. A proposed schematic model depicts how tolerable glycometabolic stress boosts cancer cell resilience through altered *N*-glycosylation and Notch signaling activation.**

**Supplementary Fig. S4. MALDI-TOF MS analysis of LLO glycans separated by HPLC.**

**A** HPLC profile of LLO glycans. **B** MALDI-TOF MS of LLO glycans corresponding to peaks I to IX in (**A**). The possible structure of each peak was predicted by GlycoWorkbench software. # indicates the sodium ion form of the major peak.
