# Supplementary figures and images for: Tolerable glycometabolic stress boosts cancer cell resilience through altered N-glycosylation and Notch signaling activation
Source: Cell Death Dis. 2024 Jan 15;15(1):53. doi: 10.1038/s41419-024-06432-z (PMC10789756; doi:10.1038/s41419-024-06432-z)

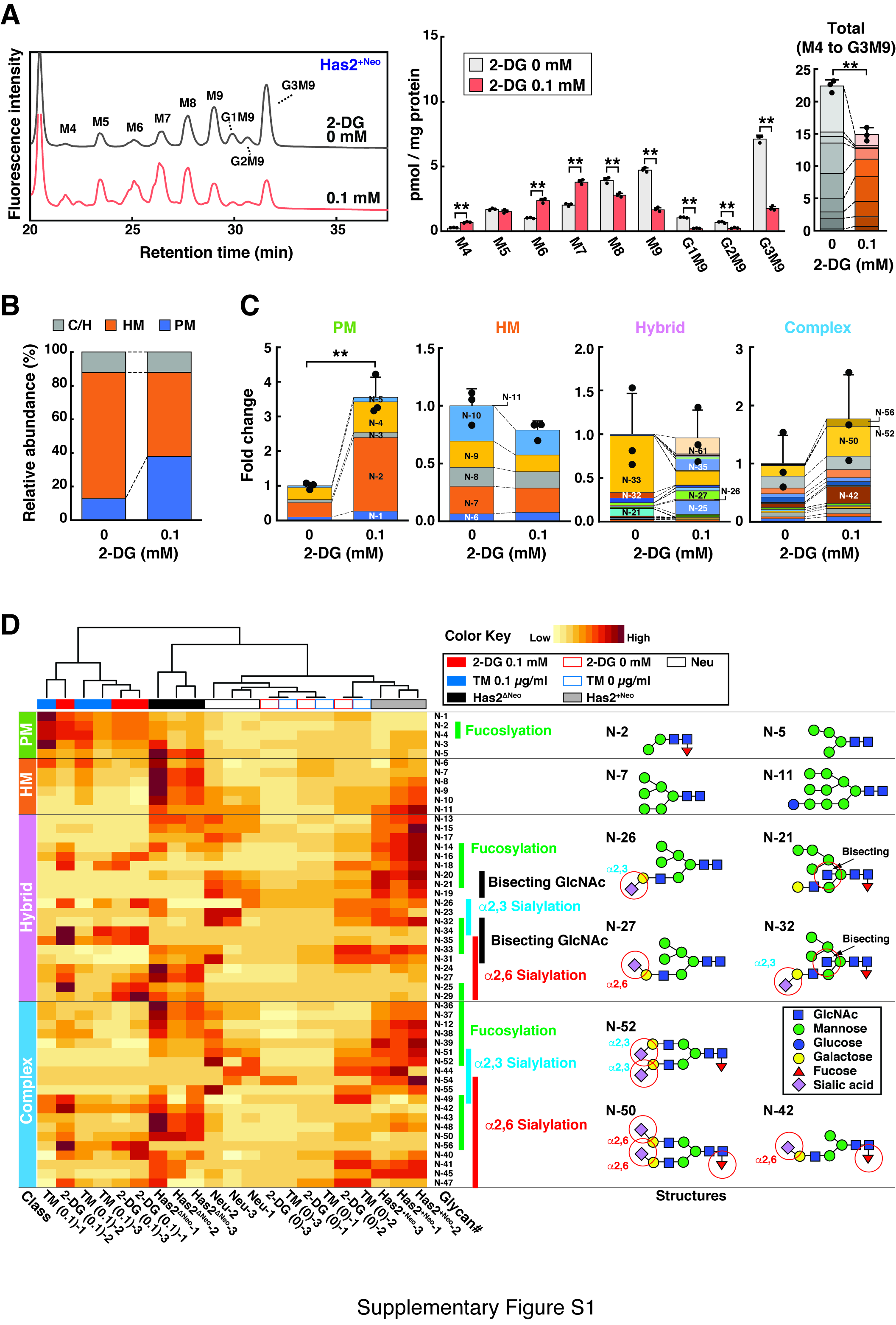

Supplement: Supplementary file 8 — Supplementary Figure S1 [file 41419_2024_6432_MOESM8_ESM.tif]

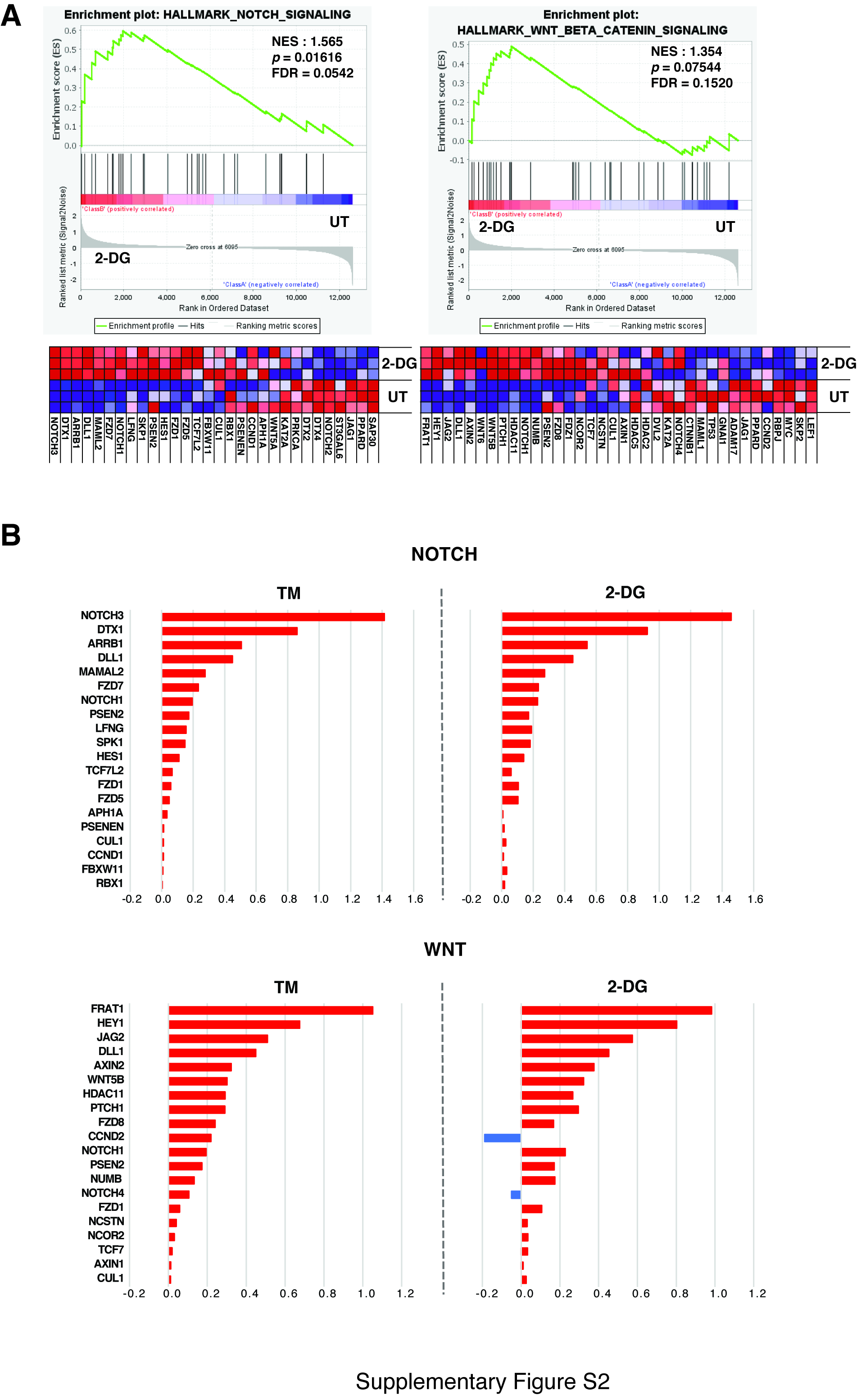

Supplement: Supplementary file 9 — Supplementary Figure S2 [file 41419_2024_6432_MOESM9_ESM.tif]

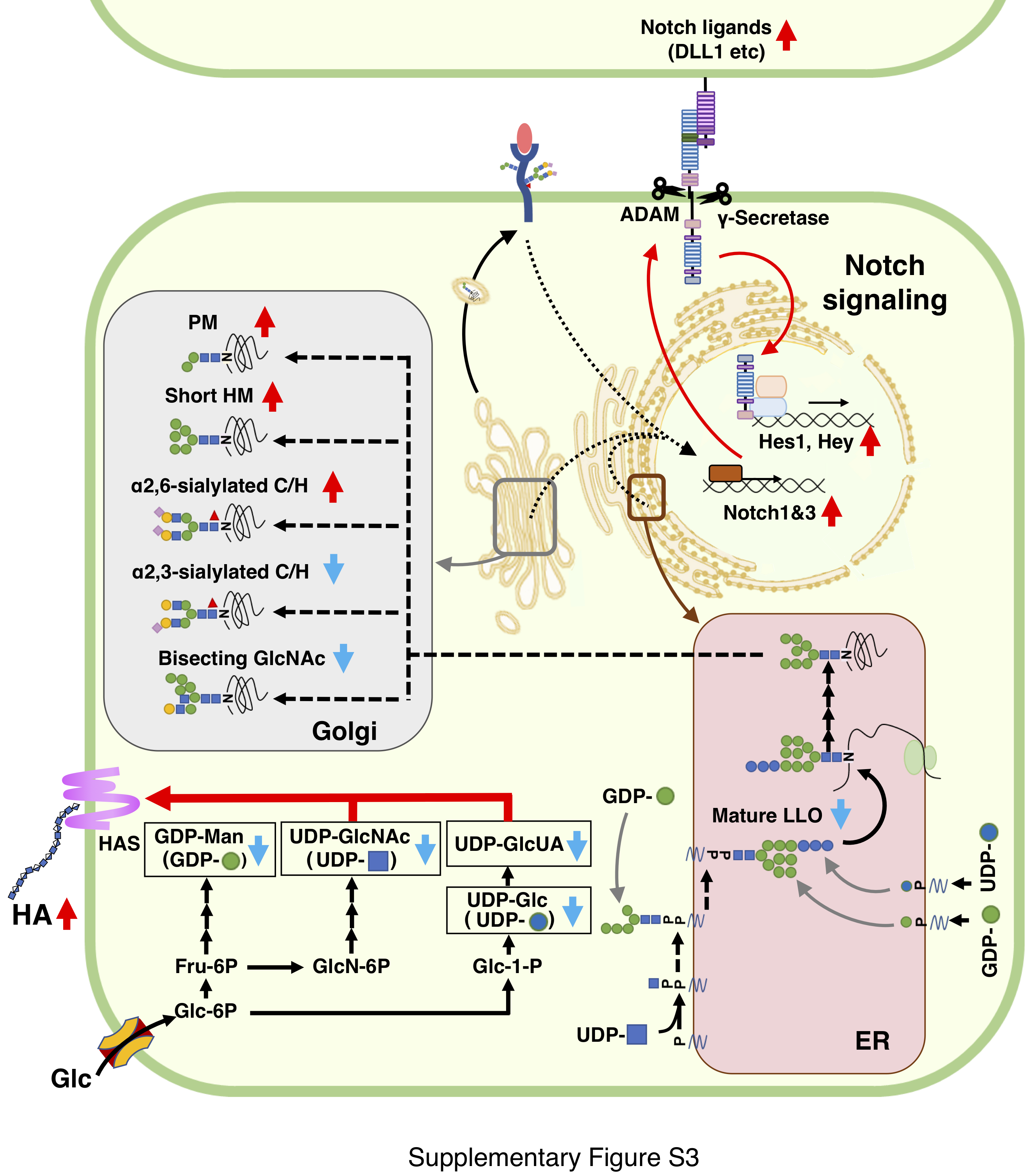

Supplement: Supplementary file 10 — Supplementary Figure S3 [file 41419_2024_6432_MOESM10_ESM.tif]

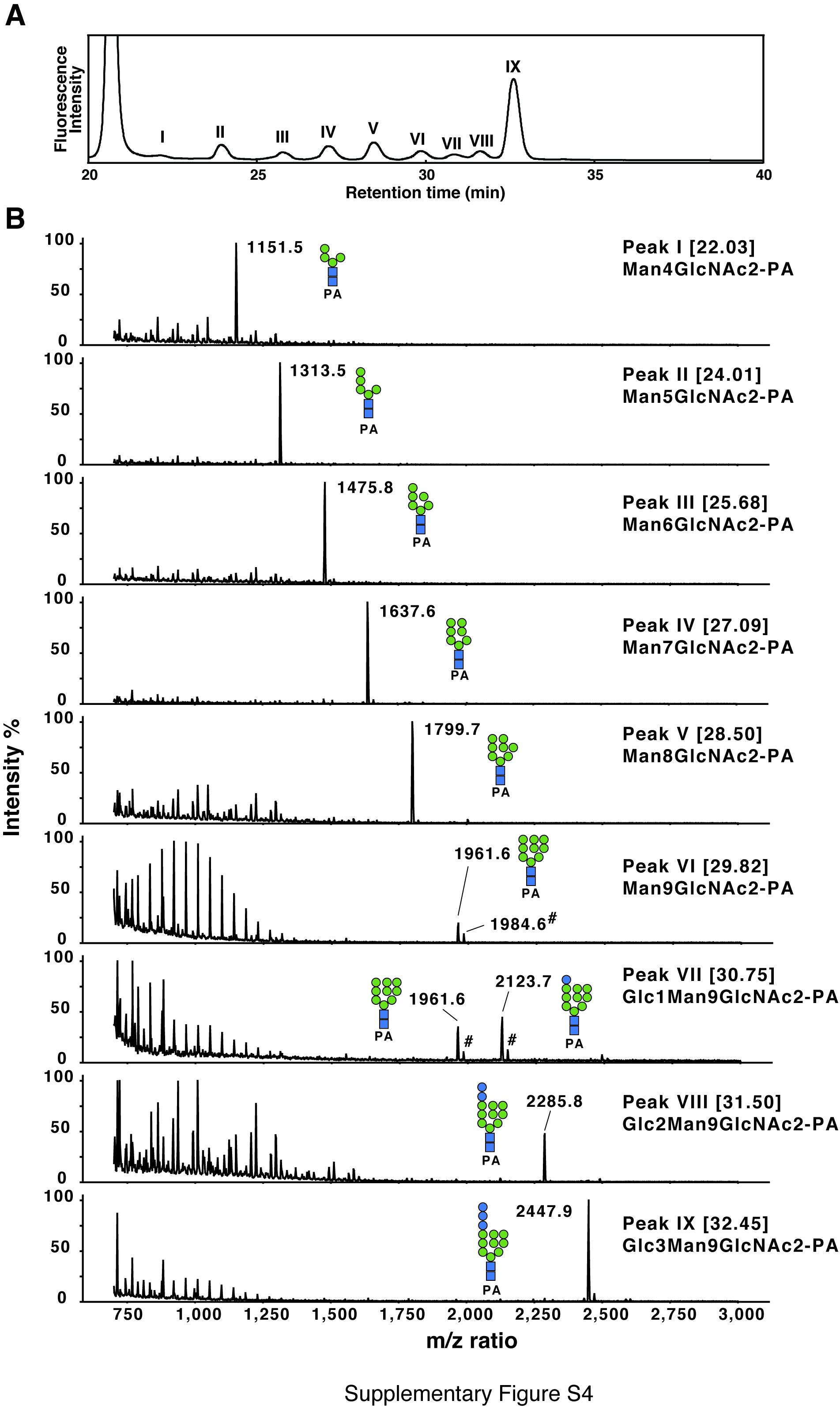

Supplement: Supplementary file 11 — Supplementary Figure S4 [file 41419_2024_6432_MOESM11_ESM.tif]
